# Composite ZIF-8 with Cs_3_Bi_2_I_9_ to Enhance the Photodegradation Ability on Methylene Blue

**DOI:** 10.3390/molecules30071413

**Published:** 2025-03-22

**Authors:** Tao Tang, Haoran Zhang, Hexu Wang, Xiaoyu Dou, Jianfeng Wen, Li Jiang

**Affiliations:** 1 Key Laboratory of Low-Dimensional Structural Physics and Application, Education Department of Guangxi Zhuang Autonomous Region, College of Physics and Electronic Information Engineering, Guilin University of Technology, Guilin 541004, China; tangtao@glut.edu.cn (T.T.); glzhanghaoran@163.com (H.Z.); wanghx0907@163.com (H.W.); douxiaoyu1018@163.com (X.D.); 2 School of Electronic Information and Automation, Guilin University of Aerospace Technology, Guilin 541004, China

**Keywords:** photocatalysis, lead-free perovskites, Cs_3_Bi_2_I_9_, ZIF-8

## Abstract

The development of green, efficient, and reusable photocatalysts is important for pollution degradation. In recent years, Cs_3_Bi_2_I_9_ has been shown to be an effective photocatalyst. However, the rapid recombination of electrons and holes weakens the photocatalytic activity. In this work, the photogenerated electron transfer rate was promoted by ZIF-8 compositing with Cs_3_Bi_2_I_9_, which effectively improved the pollutant degradation. After 50 min of visible light irradiation, Cs_3_Bi_2_I_9_/ZIF-8 removed up to 98.2% of methylene blue (MB), which was 4.15 times higher than that of Cs_3_Bi_2_I_9_ alone. In addition, the Cs_3_Bi_2_I_9_/ZIF-8 composite still exhibited high photocatalytic activity after three cycling experiments. Our research offers a simple and efficient method for enhancing the photocatalytic activity of lead-free halide perovskites.

## 1. Introduction

While actively promoting industrialization, countries have also brought tremendous challenges to environmental protection and resource conservation [1,2,3]. In recent years, the excessive use of various chemical reagents in the pharmaceutical, agricultural, and manufacturing industries has led to increasingly serious environmental pollution, which is contrary to the original intent of sustainable development [4,5,6]. Among these chemicals, methylene blue (MB) is a typical organic pollutant. Its aqueous solution is alkaline and harmful, causing serious environmental problems due to its non-biodegradation and accumulation in the environment [7]. The common strategies used to treat MB in wastewater are adsorption [8], biodegradation [9], and photocatalytic degradation [10]. Adsorption methods mainly suffer from high costs and secondary pollution issues, while biodegradation methods have long degradation cycles and strict restraint of degradation environments. In contrast, photocatalytic degradation of MB is the most efficient, environmentally friendly, and practical method.

Many materials such as TiO_2_ [11], ZnO [12], CuO [13], Fe_2_O_3_ [14], ZnS [15], and graphene [16] have been widely used for photocatalytic degradation [17,18,19]. However, wide bandgap, low visible light utilization, and fast recombination of photogenerated electron and hole pairs are potential limitations that restrict the photocatalytic activity. Recently, lead-based perovskites have been shown to be an efficient visible-light photocatalyst [20,21,22]. However, the toxicity of lead and instability of these perovskites have hindered its application in photocatalysis [23,24]. Guo et al. used a novel photocatalyst CsPbBr_3_/UiO-66 to degrade 91.6% of 10 mg/L methyl orange in 90 min under visible light [25]. Gu et al. used nitrogen-doped graphene quantum dots as surfactants to prepare δ-phase CsPbI_3_ nanocrystals and degraded 96% of rhodamine B in 4 h [26]. Although water stability can be improved by ligand exchange and surface functionalization, it is more important to develop lead-free halide perovskite photocatalysts that are non-toxic and water-stable [27]. In recent years, bismuth-based perovskites [28], which combines low toxicity with excellent water stability, have been discovered in the family of non-lead halide perovskites. In particular, a pioneering study by Sagar M. Jain et al. who chose lead-free bismuth-based perovskite (CH_3_NH_3_)_3_Bi_2_I_9_ for the preparation of solar cells, successfully converted solar energy into electric energy, achieving a conversion efficiency of 22.3%, which attracted widespread attention [29]. This breakthrough not only highlights the great potential of bismuth-based perovskites in the field of solar energy conversion but also heralds their important role as efficient photocatalysts.

Among them, inorganic halide perovskite Cs_3_Bi_2_I_9_ has attracted much attention due to its fast carrier transport, high defect tolerance, very low defect density, high water stability, and low toxicity [30,31,32]. Its photoluminescence quantum yield is 2.3% [33]. Effectively separating these electron-hole (e-h) pairs and transferring carriers is a feasible method to increase the number of carriers participating in photocatalysis [34,35,36]. Dou et al. demonstrated excellent stability by using Cs_3_Bi_2_I_9_ composited with Ti_3_C_2_ and showed more than 97.3% rhodamine B removal efficiency under visible light [37]. Therefore, it is proved feasible to find a co-catalyst to enhance the rapid separation and transfer of photoexcited e-h pairs in Cs_3_Bi_2_I_9_ to improve the photocatalytic performance [38,39]. In recent years, ZIF-8 has been used in electron transport layers due to its simple synthesis method, good conductivity, good stability, large specific surface area, high carrier mobility, and abundant active center sites [40].

In this study, the photocatalytic degradation performance of MB in aqueous solution was investigated by using Cs_3_Bi_2_I_9_/ZIF-8 composites as photocatalysts. When ZIF-8 is combined with Cs_3_Bi_2_I_9_, ZIF-8 provides abundant charge transfer channels, and the photogenerated electrons in Cs_3_Bi_2_I_9_ can be rapidly transferred to the surface of ZIF-8, which is conducive to the effective transfer and separation of photogenerated carriers [41,42,43], thus greatly improving the photocatalytic efficiency of Cs_3_Bi_2_I_9_. Under visible light, Cs_3_Bi_2_I_9_/ZIF-8 can degrade 98.2% of MB (20 mg/L) within 50 min, much higher than Cs_3_Bi_2_I_9_ (<50%), and there was almost no decrease in the photocatalytic activity after three cycles of the experiment. In addition, the fluorescence performance of Cs_3_Bi_2_I_9_/ZIF-8 photocatalyst in water remains almost unchanged for 30 days, demonstrating its good water stability. This work demonstrates that, by compositing ZIF-8 with Cs_3_Bi_2_I_9_, a highly efficient water-stable perovskite-based photocatalyst is obtained, which opens up prospects for the photocatalytic application of non-lead perovskites.

## 2. Results

**Figure 1:**
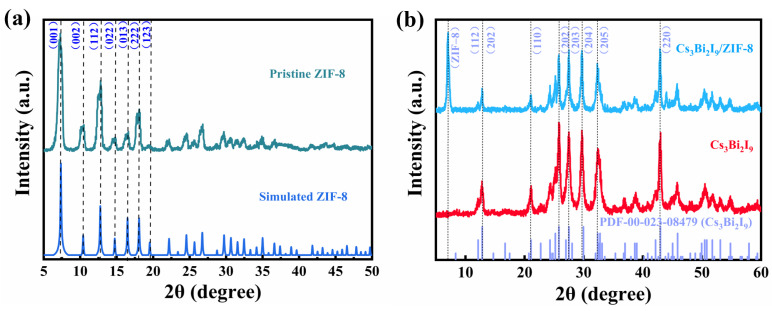
XRD patterns of (**a**) ZIF-8 and (**b**) Cs_3_Bi_2_I_9_ and Cs_3_Bi_2_I_9_/ZIF-8. The simulated and standard XRD patterns of ZIF-8 and Cs_3_Bi_2_I_9_ are used for comparison.

The crystal structures of ZIF-8, Cs_3_Bi_2_I_9_, and Cs_3_Bi_2_I_9_/ZIF-8 were analyzed by XRD. The crystal structure of ZIF-8 characterized by XRD is shown in Figure 1a. The principal diffraction peaks of ZIF-8 are located at 2θ = 7.2°, 10.2°, 12.6°, 14.7°, 16.4°, 17.9°, 19.5°, 22.1°, 24.6°, 26.8°, 29.7°, 30.6°, which are attributed to the (001), (002), (112), (022), (013), (222), and (123) crystal planes, respectively [44]. The XRD patterns of Cs_3_Bi_2_I_9_ and Cs_3_Bi_2_I_9_/ZIF-8 are shown in Figure 1b. The diffraction peaks at 2θ = 21.11°, 25.84°, 27.52°, 29.72°, 32.35°, and 42.97°, respectively, correspond to (110), (202), (203), (204), (205), and (220) crystal planes of cubic-phase Cs_3_Bi_2_I_9_ [45]. This indicates that both the synthesized ZIF-8 and Cs_3_Bi_2_I_9_ exhibit high crystallinity and are consistent with the standard XRD patterns. In the XRD pattern of Cs_3_Bi_2_I_9_/ZIF-8, the characteristic peak at 7.2° attributed to ZIF-8 can be clearly resolved, while other peaks are overlapped with those belonging to Cs_3_Bi_2_I_9_. These results suggest that ZIF-8 was successfully loaded onto Cs_3_Bi_2_I_9_.

**Figure 2:**
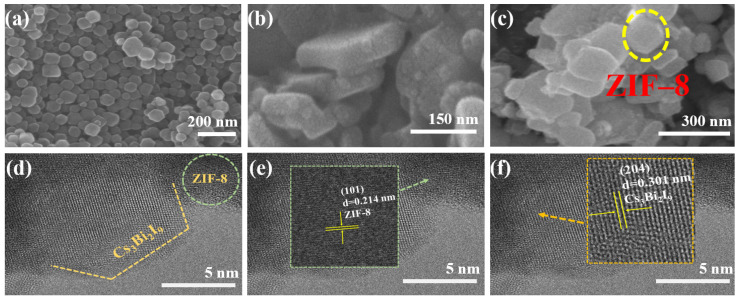
SEM images of (**a**) ZIF-8, (**b**) Cs_3_Bi_2_I_9_, (**c**) Cs_3_Bi_2_I_9_/ZIF-8. (**d**) High-resolution TEM image of Cs_3_Bi_2_I_9_/ZIF-8. High-resolution TEM images of (**e**) ZIF-8 and (**f**) Cs_3_Bi_2_I_9_ in Cs_3_Bi_2_I_9_/ZIF-8 composite.

The morphologies of the ZIF-8, Cs_3_Bi_2_I_9_ and Cs_3_Bi_2_I_9_/ZIF-8 can be clearly observed through SEM images. As shown in Figure 2a, it can be observed that ZIF-8 is a rhombic dodecahedral crystal with the particle size between 50 and 100 nm [46]. Figure 2b shows that Cs_3_Bi_2_I_9_ exhibits a hexagonal structure with a larger lateral size of 200–400 nm. In the SEM image of Cs_3_Bi_2_I_9_/ZIF-8 [Figure 2c], it is observed that small-size ZIF-8 regular particles were stacked on Cs_3_Bi_2_I_9_ platelets. Figure 2d presents the typical TEM image of Cs_3_Bi_2_I_9_/ZIF-8. The boundary of the blurred Cs_3_Bi_2_I_9_ hexagon and the irregular boundary of ZIF-8 can be discernible. Through high-resolution TEM, two different lattice stripes of Cs_3_Bi_2_I_9_ and ZIF-8 can be further clearly distinguished. The typical lattice spacings are 0.214 and 0.301 nm [Figure 2e,f], corresponding to the (101) crystal face of ZIF-8 and the (204) crystal face of Cs_3_Bi_2_I_9_, respectively [47,48].

**Figure 3:**
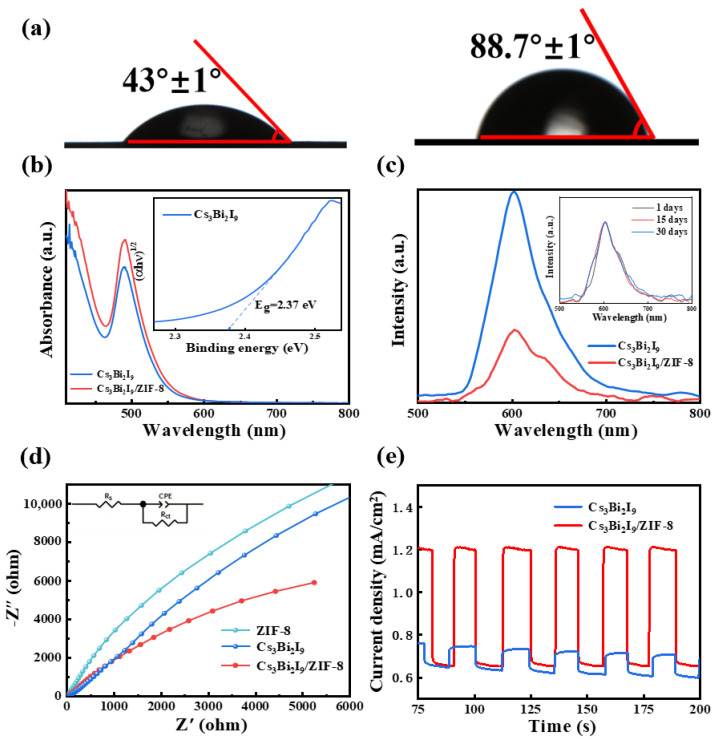
(**a**) Water contact angles of Cs_3_Bi_2_I_9_ and Cs_3_Bi_2_I_9_/ZIF-8. (**b**) UV–Vis absorption spectra of Cs_3_Bi_2_I_9_ and Cs_3_Bi_2_I_9_/ZIF-8. The inset is the corresponding Tauc plot of Cs_3_Bi_2_I_9_. (**c**) PL spectra of Cs_3_Bi_2_I_9_ and Cs_3_Bi_2_I_9_/ZIF-8. The inset is the PL spectra of Cs_3_Bi_2_I_9_/ZIF-8 on the 1st, 15th, and 30th day. (**d**) Electrochemical impedance measurements of ZIF-8, Cs_3_Bi_2_I_9_ and Cs_3_Bi_2_I_9_/ZIF-8. The inset is the equivalent circuit diagram, where R_s_, R_ct_ and CPE denote the total resistance of the external circuit, the charge transfer resistance and constant phase element. (**e**) The visible-light photocurrent responses of Cs_3_Bi_2_I_9_ and Cs_3_Bi_2_I_9_/ZIF-8.

Water stability serves as a crucial indicator in assessing the suitability of a catalyst for applications in aqueous environments. As illustrated in Figure 3a, the water contact angles corresponding to Cs_3_Bi_2_I_9_ and Cs_3_Bi_2_I_9_/ZIF-8 are 43° and 88.7°, respectively. The significantly larger water contact angle observed for Cs_3_Bi_2_I_9_/ZIF-8 suggests a reduced interaction surface with water. Thus, to some extent, it can be indirectly determined that Cs_3_Bi_2_I_9_/ZIF-8 has a better water stability. Its optical properties were studied through UV-Vis absorption and PL tests. The band gap of ZIF-8 is 4.9 eV, which is insensitive to the visible-region light [49]. In Figure 3b, the absorption properties of Cs_3_Bi_2_I_9_/ZIF-8 are identical to those of Cs_3_Bi_2_I_9_, with absorption edges up to 600 nm. Based on Tauc plots [50,51], the bandgap (E_g_) of Cs_3_Bi_2_I_9_ can be concluded to be 2.37 eV [inset of Figure 3b]. In Figure 3c, it is observed that the PL intensity of Cs_3_Bi_2_I_9_/ZIF-8 is significantly stronger than Cs_3_Bi_2_I_9_ with similar PL centers and shapes, and thus, it is demonstrated that many of the e-h pairs in Cs_3_Bi_2_I_9_ no longer recombine in an emissive manner after ZIF-8 compositing. From the inset of Figure 3c, it can be noticed that the PL shape and intensity of Cs_3_Bi_2_I_9_/ZIF-8 in water remain almost unchanged for 30 days, which indicates its excellent water stability. In addition, the arc radius of Cs_3_Bi_2_I_9_/ZIF-8 is smaller than that of ZIF-8 and pure Cs_3_Bi_2_I_9_ as measured by EIS [Figure 3d], suggesting that it possesses the lowest electron-transfer resistance, which is beneficial for the effective separation and migration of photogenerated carriers. In Figure 3e, the photocurrent response of Cs_3_Bi_2_I_9_ is enhanced after ZIF-8 compositing under the same visible illumination, again indicating an increase in conducting carriers and a decrease in radiative recombination carriers. In a word, such experiments show that after ZIF-8 compositing, more photogenerated carriers in Cs_3_Bi_2_I_9_ no longer undergo the radiative emission and can be effectively transferred to ZIF-8, which benefits their participation in the photocatalytic process.

**Figure 4:**
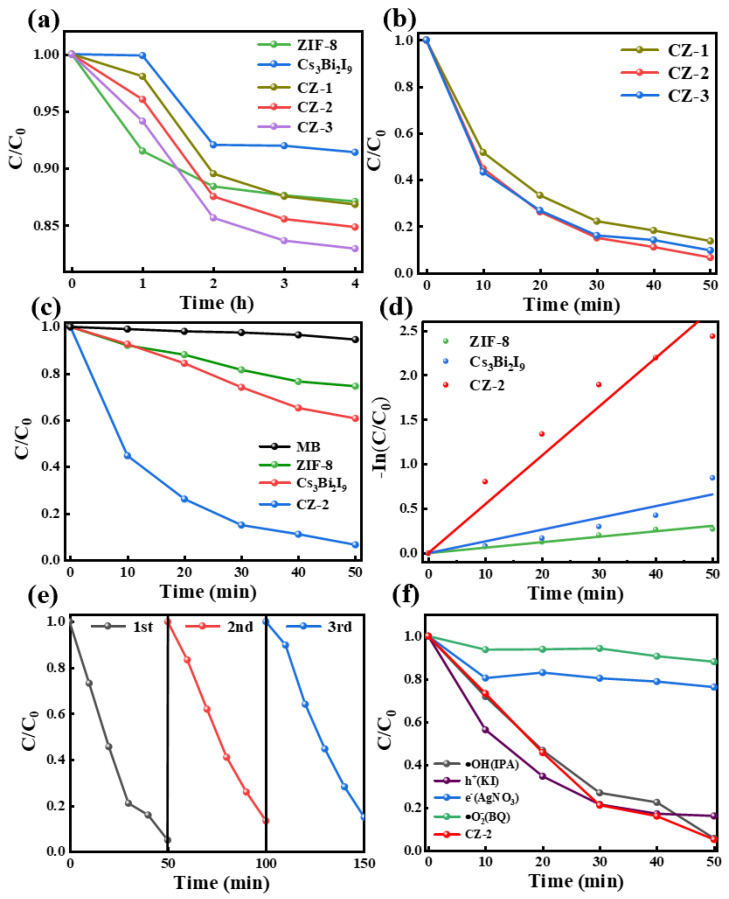
(**a**) The MB adsorption performances in the dark. (**b**) Photocatalytic degradation of MB of Cs_3_Bi_2_I_9_/ZIF-8 with different ZIF-8 mass. (**c**) The MB photodegradation of different samples and (**d**) the corresponding photodegradation kinetics behavior. (**e**) Photocatalytic cycle tests of Cs_3_Bi_2_I_9_/ZIF-8. (**f**) The impact of photocatalytic effect of Cs_3_Bi_2_I_9_/ZIF-8 after scavengers addition.

ZIF-8 is a strong adsorbent which can almost completely adsorb some organic dyes within merely tens of minutes. However, by adsorption alone, MB is very hard to separate from water [52]. The high e-h recombination rate of Cs_3_Bi_2_I_9_ gives rise to a relatively low photodegradation activity. When ZIF-8 is combined with Cs_3_Bi_2_I_9_, the resulting composite exhibits enhanced effectiveness in the decontamination process for removing MB through the adsorption–photodegradation pathway [4]. As shown in Figure 4a, for ZIF-8, Cs_3_Bi_2_I_9_ and Cs_3_Bi_2_I_9_/ZIF-8, the adsorption–desorption equilibrium in the dark can be achieved in 2 h. One can find that the MB adsorption ability of Cs_3_Bi_2_I_9_ is merely ~8% and that of ZIF-8 is ~13%, and however, that of Cs_3_Bi_2_I_9_/ZIF-8 is slightly increased (~15%), which may be attributed to the increase in specific surface area due to the uniform distribution of ZIF-8 on Cs_3_Bi_2_I_9_.

When ZIF-8 is composited with Cs_3_Bi_2_I_9_, it provides more adsorption sites, which are able to capture and immobilize the target pollutants more efficiently, thus improving the adsorption efficiency. The increasing adsorption properties of CZ-1, CZ-2, and CZ-3 were attributed to the increase in ZIF-8 content. As illustrated in Figure 4b, it is evident that under visible light conditions, CZ-1, CZ-2, and CZ-3 exhibit photodegradation efficiencies of 86.3%, 93.4%, and 90.3%, respectively. When the content of ZIF-8 increased from 20 to 30 mg, the photocatalytic efficiency actually decreased, possibly due to too much ZIF-8 blocking the effective light absorption of Cs_3_Bi_2_I_9_. As shown in Figure 4c, Cs_3_Bi_2_I_9_/ZIF-8 shows an obviously higher MB photodegradation efficiency (93.4%) than Cs_3_Bi_2_I_9_ (40%), and along with the adsorption, the total MB removal ratio of Cs_3_Bi_2_I_9_/ZIF-8 (98.2%) is undoubtedly higher than that of sole Cs_3_Bi_2_I_9_ (<50%). Compared with Cs_3_Bi_2_I_9_, the improvement in photocatalytic performance of Cs_3_Bi_2_I_9_/ZIF-8 is attributed to the effective separation and transfer of photo-generated carriers, as discussed in Figure 3. Interestingly, sole ZIF-8 exhibits very weak photodegradation ability on MB, may be due to its abundant active sites [47]. Fitting the corresponding photodegradation kinetics using a first-order reaction equation:

(1) $$ln\frac{C}{C_{0}}=-kt$$

where k is the photocatalytic efficiency (in this discussion its unit min^−1^ is omitted). As shown in Figure 4d, the k value of CZ-2 is 0.05, which is 4.15 times and 8.90 times of Cs_3_Bi_2_I_9_ (k = 0.012) and ZIF-8 (k = 0.005), respectively. In order to investigate recycling property of the photocatalyst Cs_3_Bi_2_I_9_/ZIF-8, three cycles of photocatalytic experiments were conducted. As shown in Figure 4e, in the three cycles, the photodegradation efficiencies were 97%, 89%, and 85%, respectively. There is a slight decrease because the surface of Cs_3_Bi_2_I_9_/ZIF-8 adsorbs MB molecules and it is hard to completely remove them. In order to determine the mechanism of the photocatalytic reaction, free radical trapping experiments were performed. As shown in Figure 4f, benzoquinone (BQ), isopropanol (IPA), silver nitrate (AgNO_3_), and potassium iodide (KI) were used to capture superoxide radicals (•O_2_^−^), hydroxyl radicals (•OH), electrons, and holes, respectively. There was no significant decrease in the catalytic activity of Cs_3_Bi_2_I_9_/ZIF-8 in the presence of IPA and KI. However, when in the presence of AgNO_3_ and BQ, the degradation of MB by Cs_3_Bi_2_I_9_/ZIF-8 was merely 24% and 12%, respectively, indicating that the catalytic activity decreased greatly. This indicates that •O_2_^−^ radicals and electrons were the main active substances during the photocatalytic degradation process.

In addition, Table 1 compares the relevant photocatalysts used for MB degradation in several studies. It can be seen that many photocatalysts can effectively degrade MB, but the photocatalytic time and degradation efficiency are not satisfactory. Compared with other photocatalyst materials, Cs_3_Bi_2_I_9_/ZIF-8 can photodegrade high-concentration MB with a low dosage, as well as a high degradation efficiency with a short light exposure time. Obviously, Cs_3_Bi_2_I_9_/ZIF-8 is a very promising photocatalyst.

**Figure 5:**
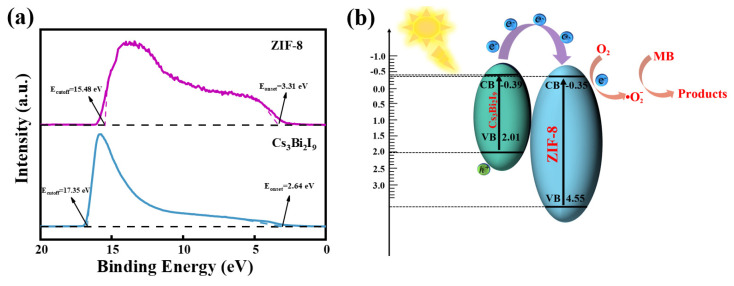
(**a**) UPS spectra of ZIF-8 and Cs_3_Bi_2_I_9_. (**b**) The mechanism of photocatalytic process of Cs_3_Bi_2_I_9_/ZIF-8.

In order to investigate the mechanism of the photocatalysis, the valence band maxima (E_VBM_) and conduction band minima (E_CBM_) of ZIF-8 and Cs_3_Bi_2_I_9_ were calculated by UPS measurements [Figure 5a] with E_VBM_ = −[21.22 − (E_cutoff_ − E_onset_)]. For ZIF-8, the E_cutoff_ was 15.48 eV and the E_onset_ was 3.31 eV. The E_VBM_ of ZIF-8 can be calculated as −9.05 eV according to the formula. Since the bandgap (E_g_) of ZIF-8 is 4.9 eV, the E_CBM_ of ZIF-8 can be calculated as −4.55 eV according to the formula E_g_ = E_CBM_ − E_VBM_ [59]. Typically, we use a standard hydrogen electrode (NHE) as a reference electrode to represent the energy levels, i.e., E_(NHE)_ = −4.5 − E_(vacuum)_, to express the relationship between the NHE and the vacuum energy levels, which results in the VBM and CBM values of 4.55 eV and −0.35 eV, respectively. Similarly, the VBM and CBM values of Cs_3_Bi_2_I_9_ were 2.01 eV and −0.39 eV, respectively, according to Figure 5a. Based on the above analysis, a possible mechanism for the photocatalytic removal of MB by Cs_3_Bi_2_I_9_/ZIF-8 can be proposed [Figure 5b]:

hν (on Cs_3_Bi_2_I_9_) → h^+^ + e^−^ (2)

When Cs_3_Bi_2_I_9_/ZIF-8 is exposed to visible light, a large number of electrons and holes are generated in Cs_3_Bi_2_I_9_, and the electrons in the valence band (VB) are excited to the conduction band (CB) while the holes are left in the VB. As shown in Equation (2), ZIF-8 cannot be photoexcited here due to its large band gap. Due to the close contact at the heterojunction interface between ZIF-8 and Cs_3_Bi_2_I_9_, electrons are spontaneously transferred from Cs_3_Bi_2_I_9_ (E_CBM_ = −0.39 eV) to ZIF-8 (E_CBM_ = −0.35 eV). The value of −0.35 eV is lower than the potential of O_2_/•O_2_^−^ (−0.33 eV); therefore, the light-generated electrons can react with dissolved O_2_ to form strongly oxidizing •O_2_^−^ at the interface. The superoxide radical (•O_2_^−^) preferentially oxidizes the thioether group (-S-) and amino group [-N(CH_3_)_2_] of MB, converting them into sulfoxide (-SO-) and sulfone (-SO_2_^−^) groups, thereby destroying the phenothiazine ring conjugation structure and generating open-ring intermediates. These open-ring products undergo hydroxylation and decarboxylation reactions to gradually release CO_2_, forming small-molecule acids such as formic acid and oxalic acid, ultimately mineralizing into CO_2_ and H_2_O. Since the results show that in our experiment only electrons and •O_2_^−^ radicals play an important role [Figure 4f], the photodegradation process can be represented in Equations (3) and (4).

O_2_ + e^−^ → •O_2_^−^ (3)

*MB + •O_2_^−^ → mineralization products (4)

In addition to enhancing the electron transfer in Cs_3_Bi_2_I_9_/ZIF-8, ZIF-8 can also provide abundant active sites and increase the contact surface area between the active sites and MB, thereby facilitating the photocatalytic reaction. The E_VBM_ of Cs_3_Bi_2_I_9_ is 2.01 eV, which is higher than the potential of H_2_O/h^+^ (1.99 eV), and however, the results in Figure 4f show that holes and •OH radicals barely take part in the photocatalytic process. Maybe it is because MB photodegradation is more likely to occur on the ZIF-8 surface of Cs_3_Bi_2_I_9_/ZIF-8.

## 3. Experimental Sections

### 3.1. Chemicals

Cesium acetate (CH_3_COOCs, 99.9%), 1-octadecene (ODE, 90%), iodotrimethylsilane (TMS-I, 97%), silver nitrate (AgNO_3_, 99.99%) and potassium iodide (KI, 98%) were purchased from Roan (Dundee, UK). Bismuth acetate [Bi(CH₃COO)₃, 99.99%] was purchased from Bidepharm (Shanghai, China). Oleic acid (OA, AR) was purchased from Aladdin (Tokyo, Japan). Oleylamine (OLA, 70%) and benzoquinone (BQ, 97%) were purchased from Macklin (Shanghai, China). Hexane (C_6_H_14_, 97%, AR), isopropyl alcohol (IPA, 99.7%, AR), sodium sulfate anhydrous (Na_2_SO_4_, 99%, AR), ethanol anhydrous (C_2_H_5_OH, 99%, GR), acetone (CH_3_COCH_3_, 99.5%, AR), and MB trihydrate were purchased from Xilong Scientific (Shantou, China). Zinc nitrate hexahydrate [Zn(NO_3_)_2_·6H_2_O] was purchased from Xilong Scientific. 2-methylimidazole (2-MIM, C_4_H_6_N_2_) was purchased from Macklin. Nafion solution (5%) was purchased from Chengxin Science and Technology (Beijing, China). The purified water was sourced from Wahaha (Hangzhou, China). All reagents and solvents were used as received without any further purification.

### 3.2. Synthesis of ZIF-8, Cs_3_Bi_2_I_9_ and Cs_3_Bi_2_I_9_/ZIF-8

Synthesis of ZIF-8: First, 0.66 g (8.04 mmol) 2-MIM was added to 14.3 mL (0.353 mol) methanol. Then, 0.3 g (1.008 mmol) Zn(NO_3_)_2_·6H_2_O was added to 14.3 mL (0.353 mol) methanol, and the solution of 2-MIM in methanol was poured into the methanol solution of Zn(NO_3_)_2_·6H_2_O. The final mixture was stirred at 1000 rpm for 1 h and then allowed to stand for 24 h. After standing, the solution was centrifuged at 8000 rpm for 3 min. Subsequently, the precipitate was washed twice with methanol, centrifuged, and dried to obtain ZIF-8.

**Figure 6:**
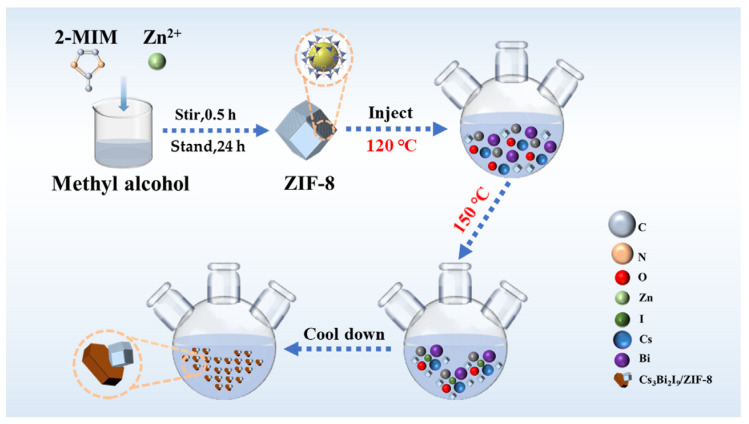
The synthesis process of Cs_3_Bi_2_I_9_/ZIF-8.

Synthesis of Cs_3_Bi_2_I_9_ and Cs_3_Bi_2_I_9_/ZIF-8: A total of 0.14 mmol cesium acetate, 0.2 mmol bismuth acetate, 1.5 mL (4.75 mmol) OA, and 0.37 mL (1.125 mmol) OLA were dissolved in 6 mL (0.01875 mol) ODE. The mixture was heated to 120 °C under N_2_ atmosphere. After maintaining the temperature for 30 min, the solution temperature was raised to 150 °C and 0.2 mL (1.7 mmol) TMS-I was rapidly injected to react for 1 min. After that, it was cooled in an ice water bath for 30 s. It was centrifuged at 8000 rpm for 15 min to obtain the precipitate. The precipitate was dispersed in hexane and centrifuged at 5000 rpm for another 15 min, and finally the precipitate was collected and dried in an oven at 60 °C for 24 h to obtain Cs_3_Bi_2_I_9_ powder. The synthesis process of Cs_3_Bi_2_I_9_/ZIF-8 is similar and shown in Figure 6, only differing in the fact that ZIF-8 was added into the cesium acetate/bismuth acetate solution when the temperature was heated to 120 °C. The obtained Cs_3_Bi_2_I_9_/ZIF-8 samples by adding 10, 20, and 30 mg ZIF-8 are, respectively, named as CZ-1, CZ-2 and CZ-3. If not specially noticed, Cs_3_Bi_2_I_9_/ZIF-8 below stands for CZ-2.

### 3.3. Adsorption and Photocatalytic Test

The adsorption capacity of the sample was evaluated in the dark. First, add 20 mg sample (ZIF-8, Cs_3_Bi_2_I_9_ or Cs_3_Bi_2_I_9_/ZIF-8) to 50 mL MB solution (20 mg/L) and stir at a certain speed. At intervals of 1 h, 3 mL of the solution is withdrawn, and the concentration of MB was quantified using a UV-Vis spectrophotometer (Shimadzu UV-2700, Kyoto, Japan) by measuring its absorbance at 664 nm. The adsorption–desorption equilibrium of all the samples could be reached in 2 h. Visible-light photodegradation of MB (50 mL, 20 mg/L) was performed under xenon lamp (PLS-SXE 300, PerfectLight, Beijing, China, 300 W, λ > 420 nm). After the adsorption–desorption equilibrium was achieved, 3 mL of solution was withdrawn each time to measure the current concentration of MB at intervals of 10 min. The MB removal (including adsorption and photodegradation) efficiency was calculated with the formula [26]

$$\mathrm{Removal}\;\mathrm{efficiency}\;(\%)=\frac{C_{0}-C}{C_{0}}\times 100\%=\frac{A_{0}-A}{A_{0}}\times 100\%$$

where C_0_ and C are the initial and real-time concentration of MB, respectively. A_0_ and A represent the initial and real-time absorbance of MB at the wavelength of 664 nm, respectively. The recycling photodegradation tests were conducted by repeatedly collecting the photocatalysts through centrifugation at 8500 rpm and drying at 60 °C. Free radical capture experiments were conducted using IPA, AgNO_3_, KI, and BQ as scavengers to determine the photocatalytic active species.

### 3.4. Electrochemical Tests

Transient photocurrents and electrochemical impedances were measured using a standard three-electrode electrochemical workstation (CHI 660E, Shanghai CH Instrument Company, Shanghai, China). The electrolyte was Na_2_SO_4_ solution (0.2 M), and the light source was a xenon lamp (PLS-SXE300, 300 W). Graphite electrode was used as the counter electrode, AgCl electrode as the reference electrode, and the sample as the working electrode. The photocatalyst was deposited onto the fluorine-doped tin oxide (FTO) electrode. A pristine FTO electrode was acquired through sequential soak and sonication in acetone, followed by deionized water washing and sonication in anhydrous ethanol. The mixture of 10 mg sample, 100 μL deionized water, 100 μL anhydrous ethanol and 30 μL nafion was sonicated for 20 min. Subsequently, the mixture was dropwise added using a pipette gun, consequently leading to the formation of an orange-red coating on the electrically charged side of the FTO electrode. After the resultant FTO electrode was dried in an oven, the sample-coating FTO electrode was then used for tests.

### 3.5. Characterization

The X-ray diffractometer (XRD, Miniflex 600, Rigaku, Tokyo, Japan) was used to study the crystal structure of Cs_3_Bi_2_I_9_, ZIF-8 and the composite material Cs_3_Bi_2_I_9_/ZIF-8. Its scanning angle ranged from 5 to 60°, with a scanning speed of 2°/min. The elemental compositions of ZIF-8, Cs_3_Bi_2_I_9_ and Cs_3_Bi_2_I_9_/ZIF-8 were characterized using an X-ray photoelectron spectrometer (XPS, Thermo Scientific K-Alpha, Waltham, MA, USA). The morphology of ZIF-8 was analyzed using scanning electron microscopy (SEM, TESCAN MIRA LMS, Brno, Czech Republic). The microstructures of Cs_3_Bi_2_I_9_ and Cs_3_Bi_2_I_9_/ZIF-8 were characterized and the lattice spacing was determined using high resolution transmission electron microscope (TEM, JEM-2100F, Hitachi, Tokyo, Japan). The valence electron structure of Cs_3_Bi_2_I_9_ was measured using UV photoelectron spectroscopy (UPS, ESCALAB 250Xi, Thermo Scientific, Waltham, MA, USA). The UV-Vis absorption spectra of photocatalysts and organic pollutants were measured using a UV spectrophotometer (UV2700, Shimadzu, Kyoto, Japan), with the measurement wavelength range being 200~800 nm. The photoluminescence (PL) spectrum of the sample was measured using a fluorescence spectrometer (Edinburgh FL/FS900 Carry Eclipse, USA) from Agilent Technologies. An excitation wavelength of 380 nm is used, and the collected emission rage is 400–800 nm. The contact angle between Cs_3_Bi_2_I_9_, Cs_3_Bi_2_I_9_/ZIF-8 and water was measured by using SDC-200 angle measuring instrument of Dongwan Shengding Precision Instrument, Dongguan, China.

## 4. Conclusions

In summary, Cs_3_Bi_2_I_9_/ZIF-8 photocatalysts with high catalytic activity were successfully prepared. The experimental results showed that the optimized Cs_3_Bi_2_I_9_/ZIF-8 had a synergistic adsorption and photodegradation removal rate of MB up to 98.2%, which was better than most materials reported previously. The excellent photocatalytic performance is attributed to the efficient separation of photogenerated e-h pairs and electron transfer in Cs_3_Bi_2_I_9_ by ZIF-8. In addition, it also advances in good water stability and reusability. The results also show that •O_2_^−^ radicals are the main substances to oxidize MB in the photocatalytic degradation process. This work provides a promising strategy in the development of stable and cost-effective photocatalysts based on lead-free halide perovskites.

## Figures and Tables

**Table 1:** Comparison of MB degradation effects of photocatalysts under visible or UV Light.

Catalyst	Illumination	Quantity	C_0_	Efficiency	Catalytic Time	Ref.
ZIF-8	Ultraviolet light	25 mg	10 mg/L	82.3%	120 min	[53]
MnTiO_3_	Visible light	25 mg	10 mg/L	89.7%	180 min	[54]
LaMnO_3_	Visible light	5 mg	5 mg/L	95%	120 min	[55]
MAIPb	Visible light	25 mg	10 mg/L	90%	60 min	[56]
BiVO_4_	Visible light	0.25 g	5 mg/L	81%	240 min	[57]
TiO_2_	Ultraviolet light	0.15 g	10 mg/L	81.4%	100 min	[58]
Cs_3_Bi_2_I_9_/ZIF-8	Visible light	20 mg	20 mg/L	93.4%	50 min	This work

## Data Availability

The original contributions presented in this study are included in the article. Further inquiries can be directed to the corresponding author.
